# Antimetastasis Effect of* Astragalus membranaceus*-*Curcuma zedoaria* via *β*-Catenin Mediated CXCR4 and EMT Signaling Pathway in HCT116

**DOI:** 10.1155/2019/9692350

**Published:** 2019-05-30

**Authors:** Xiying Tan, Mengting Xu, Fuyan Liu, Ming Xu, Yi Yao, Decai Tang

**Affiliations:** ^1^Nanjing University of Chinese Medicine, Nanjing, China; ^2^Department of Pharmacy, Affiliated Hospital of Nanjing University of Chinese Medicine, Nanjing, China; ^3^China Pharmaceutical University, Nanjing, China

## Abstract

*Astragalus membranaceus *and* Curcuma zedoaria*, two traditional Chinese medicines, are widely used together in colorectal cancer adjuvant treatment. Many different mechanisms should be involved in the benefit effect of* Astragalus membranaceus *and* Curcuma zedoaria*. In this study, we established that the combined extract from* Astragalus membranaceus *and* Curcuma zedoaria* (HQEZ) decreased the metastasis ability in colorectal cancer cells (HCT116, a cell line of colorectal carcinoma established from* Homo sapiens*)* in vitro*, and the treatment induced the downregulation of EMT signal and decreased CXCR4 expression and the level of *β*-catenin. Overexpression of CXCR4 and the administration of the agonist and inhibitor to *β*-catenin signal pathway were used to explore the mechanism of* Astragalus membranaceus *and* Curcuma zedoaria* in colorectal cancer treatment. The data demonstrated that HQEZ increased the phosphorylation of *β*-catenin which related to the degradation of *β*-catenin, and it induced the downregulation of EMT signal and CXCR4. It meant that the influence of *β*-catenin should be a key event in the antimetastasis effects of* Astragalus membranaceus-Curcuma zedoaria* in colorectal cancer model. These findings revealed the potential effect and mechanism of* Astragalus membranaceus-Curcuma zedoaria* in colorectal cancer treatment and provided insight for optimization of the usage.

## 1. Introduction

Colorectal cancer, the fourth most common cancer, affects more than one million people per year and causes more than 600000 deaths in world [1]. Most colorectal cancer patients are associating with regional or distant metastasis when they are diagnosed, and adjuvant therapies are necessary to be used after/before the surgery [1, 2]. Chemotherapy is a common adjuvant therapy which increases median overall survival especially in combination chemotherapy, whereas patients' performance status is usually not good enough to tolerate combination chemotherapy [1, 3]. Other adjuvant therapies, such as antibody therapy targeting VEGF or EGFR and immunotherapy, are also widely used and are efficacious in patients [1, 3], but the sensitivity relates to special biomarkers. Traditional Chinese medicine (TCM) is another important adjuvant therapy used to treat colorectal cancer in China and Asia for thousands of years [1, 4, 5], and their mechanisms should be explored to get more benefit effects for patients.

In cancer treatment, certain Chinese herbs or formulas, such as* Solanum incanum aqueous, Astragalus membranaceus, Curcuma zedoaria, *and* Rubus corchorifolius*, have been proved to inhibit the growth and/or metastasis of different types of cancers [4, 6, 7]. Many active ingredients separated from these herbs can inhibit the cancers directly. But Chinese herbal compound formula is the most common method used in clinic, and it is more effective when certain specific herbs and certain ratios are used together. So, researching the relationship between the compatibility and the effect may provide more information about the compatibility and/or ratio of known/unknown active ingredients.* Astragalus membranaceus* (HQ) and* Curcuma zedoaria* (EZ) are two herbs widely used in TCM to treat different cancers [8–10]. Although other TCM are also combined with these two TCM, HQ and EZ are thought to be the central herbs in many TCM prescriptions. HQ is thought to recover “Qi” which might reflect the defense to disease, and EZ is thought to break “Yu” which might reflect the abnormal aggregation. Combination of HQ and EZ increased clinic effects in the treatment of cancers, especially the colorectal cancer. As a reason, it is necessary to research the relationship between clinic effect and the compatibility of HQ and EZ in colorectal cancer treatment. Also, the potential mechanisms of the effect of HQ and EZ are necessary to be found out.

In this research, we evaluated the influence on the metastasis and growth of colorectal cancer cells with different ratios and different concentrations of HQ and EZ, which might provide a reference for clinical use. We also researched the influence on the metastasis pathways, which might reflect the mechanisms of the treatment of HQ and EZ, and it might be useful to the usage of HQEZ.

## 2. Materials and Methods

### 2.1. Reagents and Materials

HCT116 cells are purchased from FuHeng Biology (Shanghai, China). The vector overexpressing CXCR4 (MG50621-UT) was purchased from Sino Biological.* Astragalus membranaceus* (HQ) and* Curcuma zedoaria* (EZ) were purchased from Jiangsu Province Hospital of TCM. WAY-262611 (HY-11035) and SB 216763 (HY-12012) were purchased from MedChemExpress USA. Other agents were purchased from Sigma-Aldrich.

### 2.2. Cell Culture

HCT116 was cultured in complete medium (McCoy's 5A (Gibco, USA) containing 10% FBS (Bioind, Kibbutz Beit-Haemek, Israel), 100 *μ*g/ml ampicillin and 100 *μ*g/ml streptomycin) at 37°C with 5% CO2 and 95% air.

### 2.3. Preparation of Extraction of* Astragalus membranaceus* and* Curcuma zedoaria*

3 different rates of* Astragalus membranaceus* and* Curcuma zedoaria* were used to be extracted with 100 ml water for 1 hour at 100°C. The solution was concentrated to 10g crude drugs/ml. After the solution was cooled, the solution was centrifuged at 12000g for 10min and the supernatant was filtered through 0.22*μ*m filter to remove the bacteria. The filtered solution was separated and stored at -20°C. The used concentrations are represented by the weight of crude drugs per ml.

### 2.4. Cell Viability Assay [11]

5×10^3^ cells/well HCT116 were plated into 96-well plates and treated with HQEZ 12 hours later. After 24 hours, cell viability was assessed by the CCK8 assay according to the user manual. Briefly, 10*μ*l/well CCK8 was added to the 96-well plates and cultured for another 1 hour, then the optical density (OD) was measured at 450 nm on a multifunction microplate reader. The proliferation inhibition rate was calculated as per the following equation: (1-OD treated/ OD control)×100%.

### 2.5. Transwell Assay [12]

1×10^5^ cells/well HCT116 in culture medium containing 1% FBS were performed in 24-well transwell chambers (Costar). The upper surface of polycarbonate filters with 8mm pore was coated or uncoated with 50*μ*l Matrigel (Corning). The bottom chambers were added with culture medium containing 10% FBS. Cells were incubated for 48 hours. Migration or invasion was terminated by removing cells from upper chambers and stained with Crystal Violet.

### 2.6. Western Blot Analysis

Western bolt analysis was performed as described previously. The primary antibodies were as follows: rabbit anti-CXCR4 (1:500, abway), rabbit anti-E-cadherin (1:500, abway), rabbit anti-N-cadherin (1:500, abway) and anti-Vimentin (1:500, abway), rabbit anti-*β*-catenin (1:500, abway), rabbit anti-Myc (1:500, abway), rabbit anticyclin D1 (1:300, abway), rabbit anti-p-*β*-catenin (1:500, CST), rabbit anti-ERK1/2 (1:500, CST), rabbit anti-pERK1/2 (1:500, CST), rabbit anti-AKT(1:500, CST), and rabbit anti-pAKT(1:500, CST). An internal control was performed using mouse anti-GAPDH (1:2000; KangChen Bio-tech) or mouse anti-*β*-actin (1:2000; KangChen Bio-tech), and appropriate horseradish peroxidase-linked secondary antibodies were used for enhanced chemiluminescence detection (Pierce). Number of cells reaching the lower surface of the filter was counted in five random fields per filter.

### 2.7. RNA Purification, cDNA Synthesis, and Quantitative Real-Time PCR

5×10^5^ cells/well HCT116 were plated into 6-well plates and treated with HQEZ 12 hours later. After 24 hours, total RNA was purified by Trizol (15596026, Thermo Fisher) following the manuscript's protocol. And cDNA was synthesized with M-MuLV First Strand cDNA synthesis Kit (B532435, Sangon Biotech (Shanghai) Co., Ltd. China). We utilized qPCR Mastermix (B630004, Sangon Biotech (Shanghai) Co., Ltd. China) and followed its protocol to perform qPCRs in triplicate. We performed the Ct method to quantify and normalize the target mRNA expression to *β*-actin expression. The following are the primers used: *β*-actin (forward: AGCGAGCATCCCCCAAAGTT, reverse: GGGCACGAAGGCTCATCATT), CXCR4 (forward: ACTACACCGAGGAAATGGGCT, reverse: CCCACAATGCCAGTTAAGAAGA), and *β*-catenin (forward: AAAGCGGCTGTTAGTCACTGG, reverse: CGAGTCATTGCATACTGTCCA).

### 2.8. Statistical Analysis

Experiments were repeated at least three times independently. All of the data are presented as means ± standard deviation. Differences were evaluated using Student's t test, with* p*<0.05 considered statistically significant.

## 3. Results

### 3.1. HQEZ Inhibits Colorectal Cells

**Figure 1:**
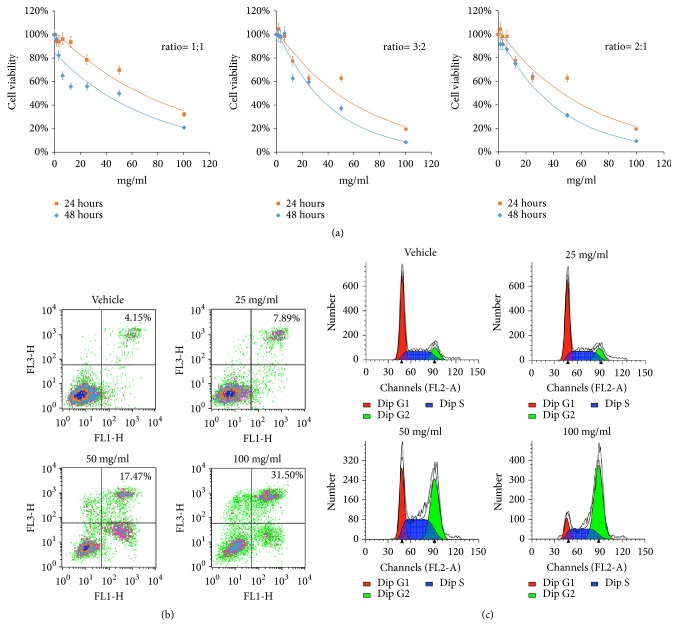
HQEZ induced cell damage of HCT116 cells. (a) In CCK-8 assay, HQEZ induced HCT116 cell damage with ratio, concentration, and time. (b) HQEZ induced apoptosis of HCT116 cells after a 48 hours treatment by flow cytometry. (c) Treated HCT116 cells with HQEZ induced cell cycle arrest.

After the incubation of 24 hours,* Astragalus membranaceus* and* Curcuma zedoaria* (HQEZ) dose-dependently inhibited the cell viability of HCT116 by CCK8 assay, and extracts from the mixture of* Astragalus membranaceus* and* Curcuma zedoaria* (2:1, weight ratio) showed the best effect on the inhibition of colorectal cells (Figure 1(a)). Also, HQEZ time-dependently inhibited the cell viability (Figure 1(b)). So we chose this ratio to further research the mechanism of HQEZ on colorectal cells.

The rate of the positive ratio of Annexin V or PI was significantly increased in high concentration of HQEZ treated HCT116 cells comparing with control group by flow cytometry (Figure 1(c)). In cell cycle assay, the inhibition of cell cycle was observed after the administration of HQEZ, and HQEZ should induce cell damage related to a G2/M arrest (Figure 1(c)). These results meant that the administration of HQEZ could induce the colorectal cancer cells apoptosis directly in a high concentration and inhibit the colorectal cancer cells viability in a relatively low concentration.

To make sure the protocol of extraction works well, we established a method of HPLC to analyze the chemical constituents of extraction of HQEZ [13]. We found the method that boiled HQ and EZ together got more Astragaloside, Astragaloside I, Astragaloside II, Calycosin, Formononetin, Curcumol, Curdione, and Germacrone than other protocols, and we could also track different batches of drugs [13].

### 3.2. HQEZ Decreases Metastasis Ability of Colorectal Cancer Cells

**Figure 2:**
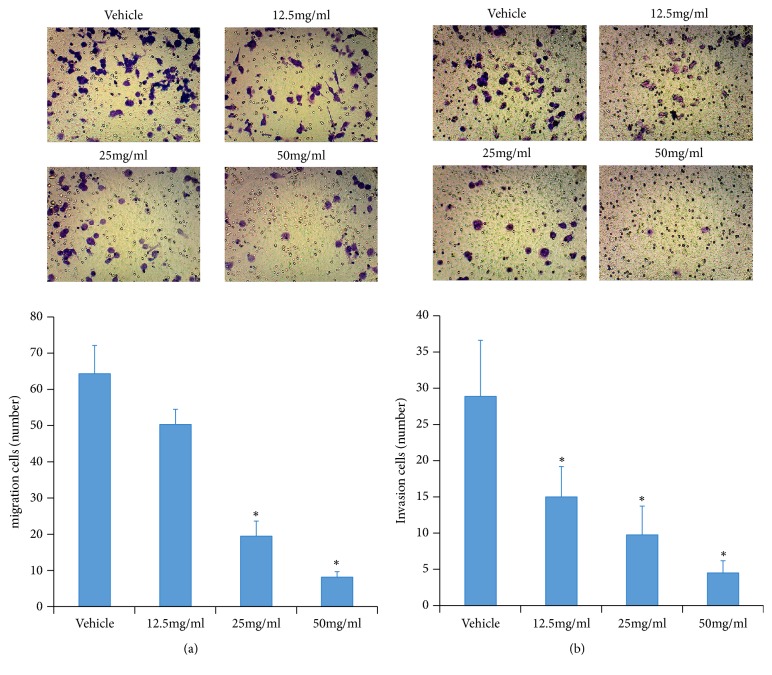
HQEZ decreases metastasis ability of HCT116 cells. (a) In a low concentration which did not induce apoptosis, HQEZ decreased the migration and (b) invasion of HCT116 cells *∗*,* p*<0.05 compared with vehicle.

Inhibited cell viability, increased apoptosis, and arrested cell cycle were observed after the administration of HQEZ, but HQEZ is usually used to decrease metastasis of colorectal cancer and prolong the life of patients in adjuvant therapies. The clinic dose of HQEZ is relatively low, so we focused on the influences of HQEZ on the metastasis ability of colorectal cells. In migration assay, HQEZ significantly decreased the migration of HCT116 cells* in vitro* (Figure 2(a)). Similar results were also observed in invasion assay that HQEZ significantly reduced the ability of invasion of HCT116* in vitro* (Figure 2(b)). In these experiments, a relatively low concentration of HQEZ, which was lower than the concentration inducing apoptosis or cell cycle arrest, decreased the metastasis ability of colorectal cells. So HQEZ could decrease the metastasis ability of colorectal cancer cells independent on the cell damage.

### 3.3. Decreased EMT Signal, CXCR4 Signal, and *β*-Catenin Signal Are Related to the Inhibition of HQEZ on Colorectal Cancer Cells

**Figure 3:**
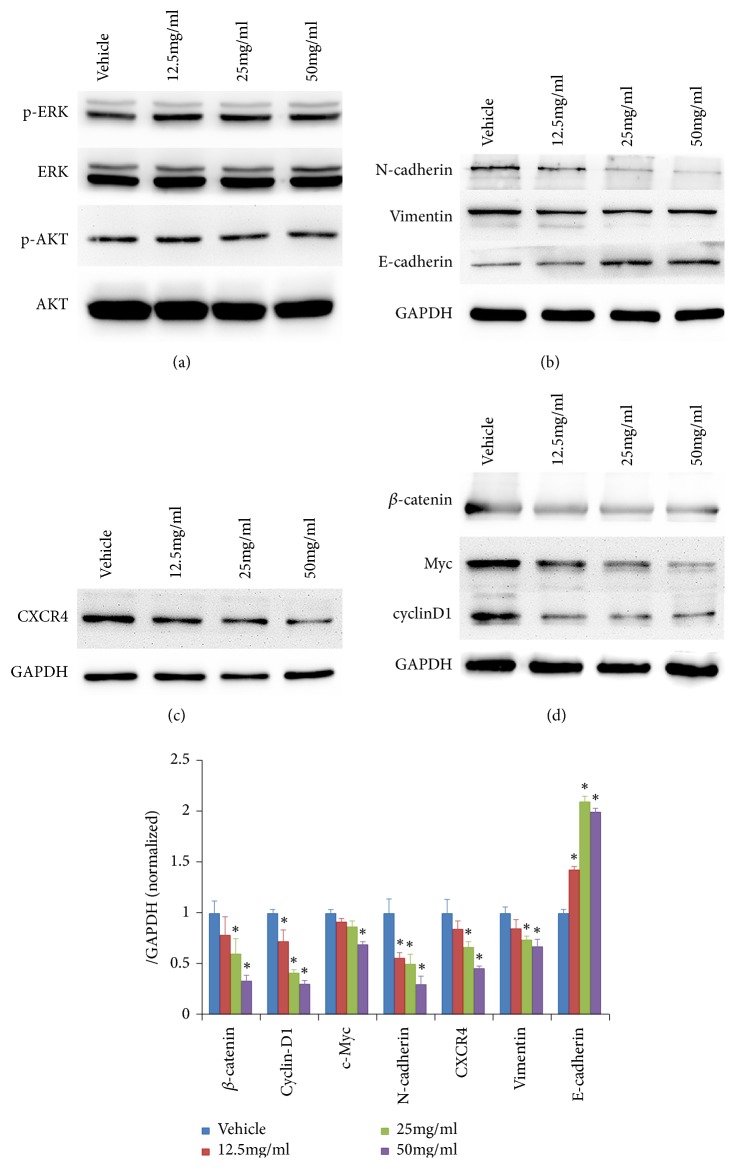
Decreased EMT signal, CXCR4 signal, and *β*-catenin signal are related to the inhibition of HQEZ on HCT116 cells. (a) The activation of AKT and ERK was not affected by the administration of HQEZ. (b) The administration of HQEZ inhibited the EMT signaling pathway which relates to the metastasis. (c) The administration of HQEZ reduced the expression of CXCR4, an important receptor mediating liver metastasis. (d) The administration of HQEZ induced degradation of *β*-catenin and the downregulation of its downstream factors. *∗*,* p*<0.05 compared with vehicle.

Many signal pathways and molecules are related to metastasis of cancers. AKT and ERK are two central factors related to metastasis and survival of cancer cells, but the treatment of HQEZ in a low concentration influenced neither the expression nor phosphorylation of AKT and ERK (Figure 3(a)). These data meant the antimetastasis effect of HQEZ should be independent on AKT or ERK signal in HCT116.

Then, EMT, another important signal related to the metastasis of cancer cell, was focused on and it was significantly inhibited by the treatment of HQEZ (Figure 3(b)). So, it reflected the possibility that the administration of HQEZ inhibited the metastasis ability of HCT116 through the inhibition of EMT signal.

Liver metastasis is a very important characteristic of colorectal cancer, and it is believed that the CXCR4 expressed on the colorectal cancer cells responds to the CXCL12 released from liver which induced the directional metastasis. After the administration of HQEZ, the expression of CXCR4 was analyzed in the HCT116, and it was decreased after the administration of HQEZ (Figure 3(c)). So we believed that the decrease of metastasis ability of HCT116, especially the liver metastasis ability, should be associated with the inhibition of CXCR4 by HQEZ.

*β*-catenin, a key factor in Wnt/*β*-catenin pathway which plays important role in the metastasis of cancers, was associated with CXCR4 activation and was also reported to influence EMT signaling pathway. After the administration of HQEZ, *β*-catenin was significantly decreased (Figure 3(d)). It meant the loss function of *β*-catenin and its downstream signal should also relate to the effect of HQEZ.

CXCR4, *β*-catenin, and EMT signaling pathway, all of which are related to each other, were inhibited by the administration of HQEZ; the relationship of these signaling pathways to HQEZ should be explored.

### 3.4. *β*-Catenin Was the Key Factor Related to the Effect of HQEZ

**Figure 4:**
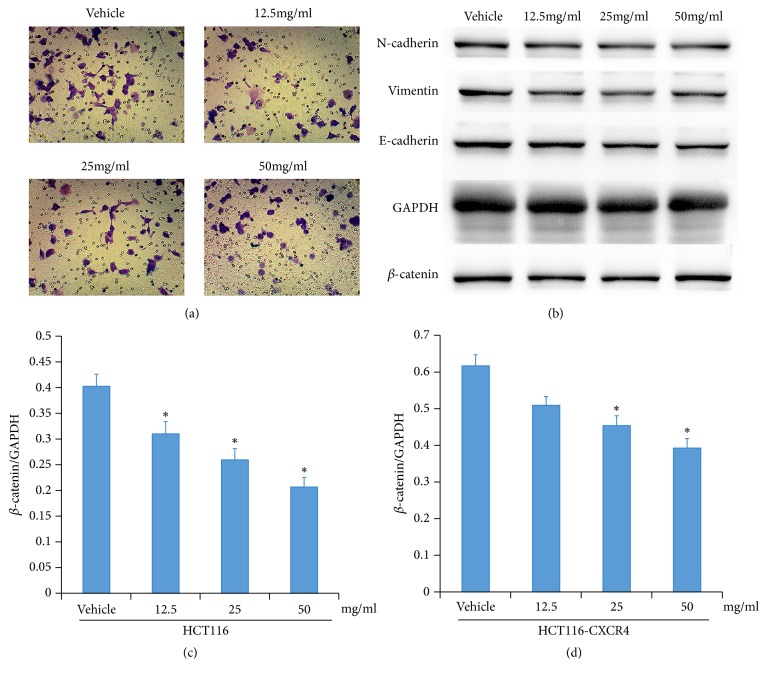
Overexpression of CXCR4 partly rescued the HCT116 cells from HQEZ. (a) Overexpression of CXCR4 rescued the metastasis ability of HCT116 cells. (b) Overexpression of CXCR4 partly rescued the EMT signal pathway and *β*-catenin. (c, d) In wild-type HCT116 and CXCR4 overexpressed HCT116, administration of HQEZ did not influence the mRNA level of *β*-catenin.

According to the results from other reports that CXCR4 could be the key factor related to metastasis effects of colorectal cancer, so we overexpressed the CXCR4 in HCT116 cells. Colorectal cancer cells overexpressing CXCR4 showed the increased metastasis ability (Figure 4(a)), but the overexpression of CXCR4 could slightly rescue the EMT signal when they were treated with HQEZ (Figure 4(b)). The decrease of *β*-catenin was also just rescued partly by CXCR4 overexpression (Figure 4(b)). The transcription of *β*-catenin was rarely decreased after the administration of HQEZ, but the transcription of CXCR4 was decreased after the administration of HQEZ (Figures 4(c) and 4(d)).

**Figure 5:**
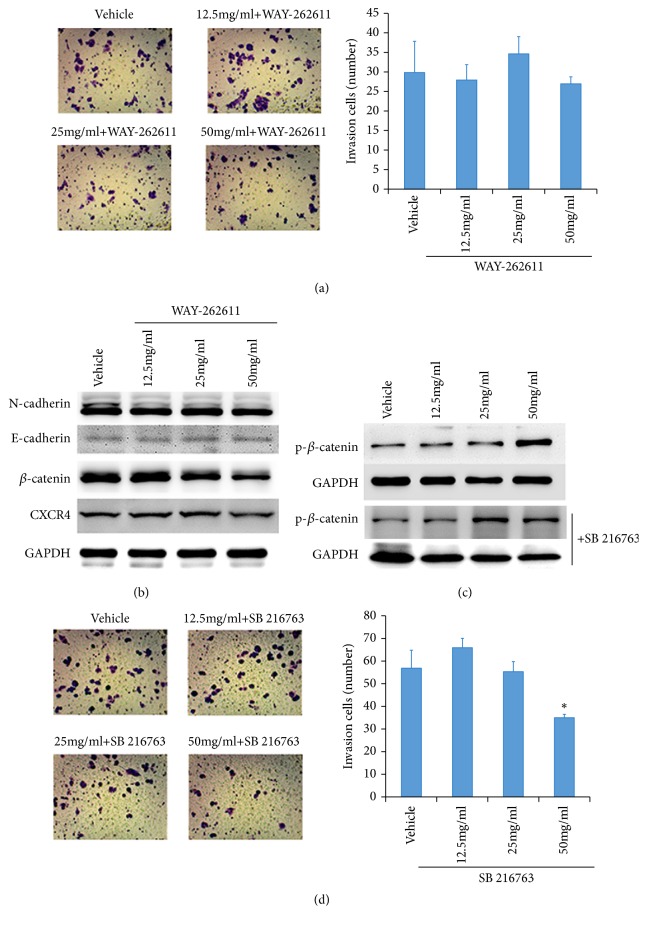
Activating Wnt/*β*-catenin pathway rescued the level of *β*-catenin, EMT signaling pathway, and the metastasis ability of HCT116 cells. (a, b) A potential activator of *β*-catenin, WAY-262611, rescued the HCT116 cells from HQEZ. (c) The degradation of *β*-catenin is related to the activity of GSK 3*β* and the phosphorylation of *β*-catenin. (d) Stabilization of *β*-catenin was important to maintain the metastasis ability. *∗*,* p*<0.05 compared with vehicle.

We speculated HQEZ influenced the *β*-catenin in a posttranscriptional level, so WAY-262611, a *β*-catenin activator, was administrated when HCT116 were treated with HQEZ. The expression of *β*-catenin was rescued by WAY-262611 (0.5*μ*M), and WAY-262611 also rescued the metastasis ability, EMT signal, and the expression of CXCR4 in HCT116 treated with HQEZ (Figures 5(a) and 5(b)). Therefore, we hypothesized that *β*-catenin might be the key factor related to the effect of HQEZ. We analyzed the phosphorylation of *β*-catenin which relates to the degradation of *β*-catenin, and we treated HCT116 with SB 216763 45min before the administration of HQEZ. According to the results, the phosphorylation of *β*-catenin was increased after the administration of HQEZ, and SB 216763 (0.1*μ*M), an inhibitor of GSK 3*β*, rescued the expression of *β*-catenin and decreased the phosphorylation of *β*-catenin (Figure 5(c)).

So, HQEZ should increase the phosphorylation of *β*-catenin with the degradation of *β*-catenin inhibiting the expression of downstream genes, which included the members in EMT signaling pathway and other factors relating to metastasis.

### 3.5. Astragalus, Curcuma, or Mixed Solution Showed Different Effects on the HCT116 Cells

**Figure 6:**
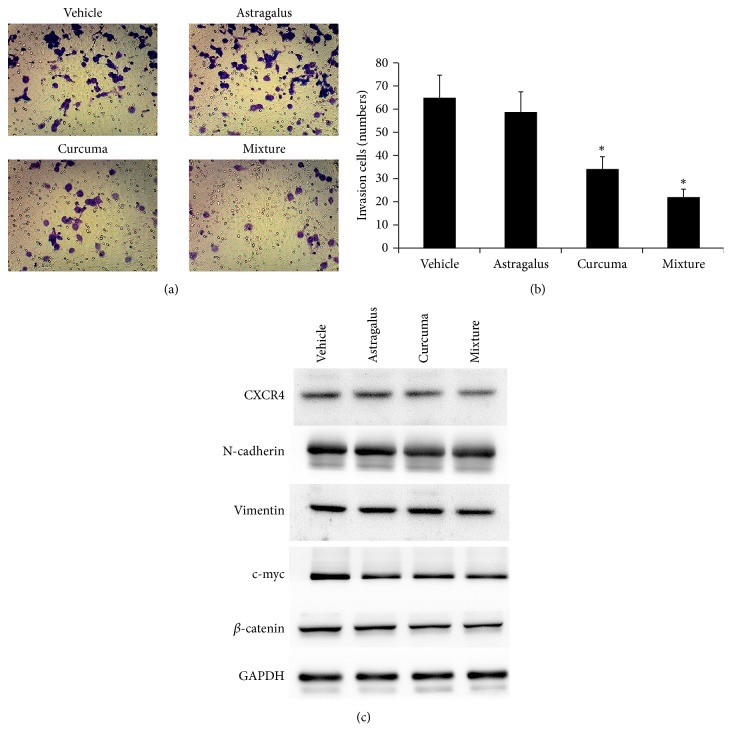
*Astragalus, Curcuma, *or mixed solution showed different effects on the HCT116 cells* in vitro*. (a, b)* Curcuma *or mixed solution but not the* Astragalus *decreased the invasion of HCT116. (c)* Curcuma *decreased CXCR4 and *β*-catenin whose effects were less than the mixture of* Astragalus and Curcuma. ∗*,* p*<0.05 compared with vehicle.

According to the results of HQEZ, HQEZ decreased the metastasis of HCT116 and this related to the inhibition of *β*-catenin mediated CXCR4 and EMT signaling pathway in HCT116. To investigate the effect of* Astragalus* and* Curcuma, *we administrated the HCT116 with* Astragalus*,* Curcuma,* or mixed solution, and we found* Curcuma* and mixed solution decreased the metastasis ability.* Curcuma* and mixed solution decreased the invasion of HCT116 (Figure 6(a)). Curcuma decreased the expression of *β*-catenin and c-myc, but the mixture decreased the expression of CXCR4 beside the *β*-catenin and c-myc (Figure 6(b)).

## 4. Discussion

*Astragalus membranaceus* is a traditional Chinese medicine widely used in cancer therapy. It is thought that* Astragalus membranaceus* restore the immune functions in cancer patients, and some natural components from* Astragalus membranaceus* enhanced chemosensitivity or inhibited cancer cells directly [14–16].* Astragalus membranaceus* also protects patients during chemotherapy against liver damage as well as nervous system damage, especially against platinum-derived toxicity [17, 18].* Curcuma zedoaria* is another traditional Chinese medicine which is usually used with* Astragalus membranaceus*, and its effects are more related to cytotoxicity and inhibition of cancers [9, 10, 19]. Here, we extracted* Astragalus membranaceus* combined with* Curcuma zedoaria* with water, which is a common usage in clinic. We found the extraction was cytotoxic in high concentration and inhibited the metastasis ability in a relatively low concentration. In adjuvant therapies, decreasing the metastasis is very important, and traditional Chinese medicines prolong the survival of patients which is partly related to the antimetastasis effect.

Activation of CXCR4, a chemokine receptor specific for stromal-derived-factor-1 (SDF-1 also called CXCL12), is highlighted in the development and the metastasis of colorectal cancers [20–22]. The SDF-1 is secreted by liver, and CXCR4 is highly expressed in the colorectal cancer cells. It is thought the CXCR4/CXCL12 axis is an important factor inducing liver metastasis in colorectal cancers [23, 24]. And the activation of CXCR4/CXCL12 axis increases the EMT [25], the expression of matrix metalloproteinases [26], and the Wnt/*β*-catenin pathway [25], all of which are important to metastasis.

We found HQEZ decreased the expression of CXCR4 in colorectal cancer cells, and we also found the inhibition of EMT and *β*-catenin. At first, we thought HQEZ should downregulate the Wnt/*β*-catenin pathway by the inhibition of CXCR4 as per previous report [11]. When we overexpressed CXCR4 in HCT116, we did not find the significant rescue of *β*-catenin or the EMT pathway, but the enhancement of *β*-catenin pathway rescued CXCR4, EMT pathway, and the metastasis ability. Some researchers reported that CXCR4 is regulated by Wnt/*β*-catenin pathway and key factors in EMT pathway are also regulated by *β*-catenin related ways. Astragaloside IV, an active component in* Astragalus membranaceus,* and *β*-elemene, an active component in* Curcuma zedoaria,* are reported to attenuate the Wnt/*β*-catenin pathway by previous reporters [14, 27]. These results are in accordance with our results that decrease the degradation of the *β*-catenin rescuing the HCT116 from HQEZ. According to these results, we thought that HQEZ induced the phosphorylation of *β*-catenin and the following degradation of *β*-catenin could induce the downregulation of EMT signal and CXCR4 which were important to inhibitive effects of HQEZ on the metastasis ability of colorectal cancer.

In conclusion, HQEZ induces the degradation of *β*-catenin in a relatively low concentration and therefore inhibits the metastasis of colorectal cancer cells* in vitro*. These results provide a potential mechanism of traditional Chinese medicine used in colorectal cancer treatment and pursue HQEZ as an adjuvant therapy used to colorectal cancer patients.

## 5. Conclusions

In this study, we evaluated the effect of* Astragalus membranaceus* and* Curcuma zedoaria* on HCT116 cells* in vitro*. When* Astragalus membranaceus* and* Curcuma zedoaria* were used at a ratio of 2:1, it showed better effects on the inhibition of HCT116 cells. At this ratio,* Astragalus membranaceus* and* Curcuma zedoaria* also showed significantly antimetastasis effect in a relatively low concentration and this effect is related to downregulation of CXCR4 and EMT signaling. These effects owned to the degradation of *β*-catenin associated with the activation of GSK-3.

## Data Availability

The data used to support our findings of this study are available from the corresponding author upon request.
